# Skin Wound Healing and Anti-Wrinkle-Promoting In Vitro Biological Activities of *Caragana sinica* Flower Absolute and Its Chemical Composition

**DOI:** 10.3390/ph16020235

**Published:** 2023-02-03

**Authors:** Mi Jung Kim, Kyung Jong Won, Do Yoon Kim, Yu Rim Won, Nan Young Kim, Da Kyoung Lee, Bok Sil Hong, Hwan Myung Lee

**Affiliations:** 1 Division of Cosmetic and Biotechnology, College of Life and Health Sciences, Hoseo University, Asan 31499, Republic of Korea; 2 Korea Essential Oil Resource Research Institute, Hoseo University, Asan 31499, Republic of Korea; 3 Department of Physiology and Medical Science, School of Medicine, Konkuk University, Seoul 05029, Republic of Korea; 4 Department of Nursing, Life Science Research Center, Cheju Halla University, Jeju 63092, Republic of Korea

**Keywords:** *Caragana sinica*, absolute, skin, wound healing, anti-wrinkle, keratinocytes

## Abstract

*Caragana sinica* (CS; family Legume) was used as a medicinal material to treat neuralgia and arthritis in folk remedies and has been shown to have antioxidant, neuroprotective, and anti-apoptotic effects. However, CS is unknown for its biological activities related to skin. The present study explored the effects of CS flower absolute (CSFAb) on skin repair responses, viz., wound healing and anti-wrinkle-related responses using keratinocytes. CSFAb was extracted using hexane, and its composition was analyzed by GC/MS. The effects of CSFAb on human keratinocytes (HaCaT cells) were evaluated using Boyden chamber, sprouting, water-soluble tetrazolium salt, 5-bromo-2′-deoxyuridine incorporation, ELISA, zymography, and immunoblotting assays. GC/MS detected 46 components in CSFAb. In addition, in HaCaT cells, CSFAb increased the proliferation, migration, and sprout outgrowth and the phosphorylation of ERK1/2, JNK, p38 MAPK, and AKT, and also increased collagen type I and IV synthesis, reduced TNF-α-increased MMP-2 and MMP-9 activities, and upregulated hyaluronic acid (HA) and HA synthase-2 levels. These effects of CSFAb on wound healing and anti-wrinkle-related responses in keratinocytes suggest its potential use for skin repair and care preparations.

## 1. Introduction

Abnormal or incomplete wound healing may damage the function of the epidermal barrier and body homeostasis [1]. Hence, many researchers are trying to develop materials that more effectively promote wound repair and restore epidermal barrier function. The wound healing process involves four complex overlapping phases, viz., hemostasis, inflammation, proliferation, and remodeling, and the precise and proper operation of these phases is closely associated with optimal wound repair [2]. These wound healing phases are orchestrated by collaboration between various cellular, biochemical, and molecular responses [3], and cellular responses during the proliferation phase are dominated by keratinocytes, the main cellular components of epidermis [4]. When the skin is damaged, keratinocytes contact collagen and initiate the repair process by migrating to wounds and proliferating [5]. Thus, keratinocyte migration and proliferation are essential events in the re-epithelialization process associated with normal repair of epidermal integrity [1,6], and these responses are activated by signaling pathways regulated by growth factors, chemokines, extracellular matrix (ECM), and metalloproteases [5]. Therefore, controlling the wound healing activities of keratinocytes offers a potential means of promoting skin wound healing and repair.

As skin ages, its functions deteriorate due to morphological and structural alterations caused by factors such as genetic and hormonal changes and sun exposure [2]. Aging-related skin changes are characterized by reduced epidermal thickness, moisture, and collagen content, which result in wrinkling and dryness [7]. Furthermore, these changes negatively affect wound healing and make skin more vulnerable to injury [2,8] and are closely associated with the activities of collagen, matrix metalloproteinases (MMPs), hyaluronic acid (HA), and hydrogen peroxide [9]. Collagen is the most abundant protein in the ECM of skin and is closely associated with its tensile strength and elasticity [9,10]. Collagen types I and III are the main interstitial structural proteins of ECM and are essential for maintaining skin tissue strength and elasticity, respectively [10]. Collagen type I is required for keratinocyte migration to wounds [11], whereas type IV collagen induces keratinocyte proliferation and migration and type VII collagen is the major basement membrane component [10,12]. ECM proteins, including type I, IV, and VII collagens, are degraded by MMPs [13,14], which also participate in cellular proliferation, migration, differentiation, and survival and tissue remodeling [13]. Furthermore, MMP-1, -2, -9, and -10 are synthesized and secreted by keratinocytes [5]. MMP-1 (a collagenase) participates in the degradation of type I and III collagens, and MMP-2 and MMP-9 (gelatinases) can hydrolyze type I and IV collagens [14]. In addition to collagens, HA is a multifunctional component of ECM that reduces skin wrinkling but increases moisture retention and regeneration [15]. HA is an ECM component, a member of the glycosaminoglycan (GAG) family, and a highly hydrophilic linear carbohydrate polysaccharide [16]. Furthermore, HA can combine with water to form a viscous material that helps hydrate and tighten skin [16]. For this reason, low skin HA contents can lead to reduced moisture contents of the stratum corneum, wrinkling, and promote skin aging [17]. HA is multifunctional and is involved in the regulations of cellular immunity, epidermal cell interactions, and the absorption of large amounts of water [18]. In addition, increased HA synthesis stimulates the wound healing migratory response of keratinocytes [19]. HA is synthesized in skin by hyaluronic acid synthase (HAS), which has three isozymes, HAS-1, HAS-2, and HAS-3 [20].

Numerous plants and plant extracts have been known to have diverse biological activities and fewer side effects than synthetic agents [21,22], and thus, they offer a valuable basis for the development of safer and more effective wound healing and anti-aging formulations. *Caragana sinica* (CS) is a deciduous shrub of the Leguminosae (or Fabaceae) family indigenous to Korea, China, and Japan [23,24]. CS has been used in traditional medicine to treat neuralgia, arthritis, and hypertension [23,24]. It has been reported that CS and its extracts have various bioactivities, including anti-bacterial, anti-inflammatory, neuroprotective, phytoestrogenic, and antioxidant properties [24,25]. However, there are no previous studies investigating the skin-associated activities of CS. Thus, in the present study, we investigated whether the absolute extracted from CS flowers (CSFAb) influences the skin wound healing and wrinkle-related responses of human keratinocytes (HaCaT cells). In addition, we subjected CSFAb to gas chromatography/mass spectrometry (GC/MS) to determine its composition

## 2. Results and Discussion

### 2.1. CS Flower Absolute-Induced Changes in the Proliferative and Migratory Activities of HaCaT Cells

**Figure 1:**
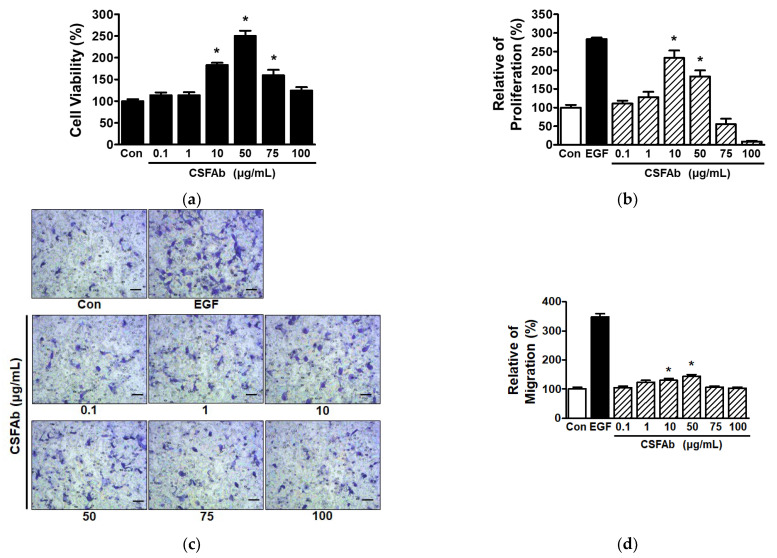
Effects of *Caragana sinica* flower absolute on the proliferation and migration of HaCaT cells. (**a**) Cell viability. HaCaT cells were incubated with or without Caragana sinica flower absolute (CSFAb; 0.1–100 µg/mL) for 48 h. Cell viabilities were evaluated using the WST assay (*n* = 4). (**b**) Cell proliferation. HaCaT cells were treated with CSFAb (0.1–100 µg/mL) for 48 h. Proliferation was assessed using the BrdU incorporation assay detailed in Section 3. Recombinant human epidermal growth factor (EGF: 50 ng/mL): the positive control. Results are expressed as mean percentages ± SEMs (*n* = 4) of untreated controls (Con). * *p* < 0.05 vs. untreated cells. (**c**,**d**) Cell migration. HaCaT cells were treated with CSFAb (0.1–100 µg/mL) for 4 h, and migrations were assessed using a Boyden chamber assay. (**c**) Representative images of experimental results. Violet spots indicate migrated cells. Scale bar = 50 μm. (**d**) Statistical graph of experimental results. Recombinant human epidermal growth factor (EGF: 1 ng/mL): positive control. Results are expressed as mean percentages ± SEMs (*n* = 4) of untreated controls (Con). * *p* < 0.05 vs. untreated cells.

To determine the effect of CSFAb on keratinocyte proliferation and migration, we first examined the cytotoxic effect of CSFAb (0.1–100 μg/mL) on HaCaT cells using a water-soluble tetrazolium (WST) assay. CSFAb induced a significant increase in HaCaT cell viability at concentrations of 10 to 75 μg/mL but had no effect at the other concentrations (Figure 1a). Thus, we used these concentrations of CSFAb in subsequent experiments. BrdU (5-bromo-2′-deoxyuridine) incorporation assays showed that CSFAb (0.1–100 μg/mL) significantly increased proliferation at 10 and 50 μg/mL (233.31 ± 19.67% and 183.72 ± 15.87%, respectively, vs. untreated controls) but did not at concentrations of 75 or 100 μg/mL (Figure 1b).

The migration of cells exposed to CSFAb (0.1–100 μg/mL) was investigated using a Boyden chamber assay. CSFAb concentration-dependently stimulated HaCaT cell migration, and this was significant at 10 and 50 μg/mL (130.51 ± 5.68% and 144.07 ± 5.95%, respectively, vs. untreated controls) (Figure 1c,d). The effect of CSFAb on HaCaT cell migration was lower than that of EGF (1 ng/mL) (348.87 ± 10.10% vs. untreated controls) (Figure 1c,d).

Keratinocytes are the major cellular component of epidermis and are essential for skin re-epithelialization by migrating to wounds and proliferating to repair injured skin [4,26]. In the present study, CSFAb was found to induce the proliferation and migration in HaCaT cells, which suggests that CSFAb might promote skin healing and repair.

### 2.2. Changes in Sprout Outgrowth of HaCaT Cells Exposed to CS Flower Absolute

**Figure 2:**
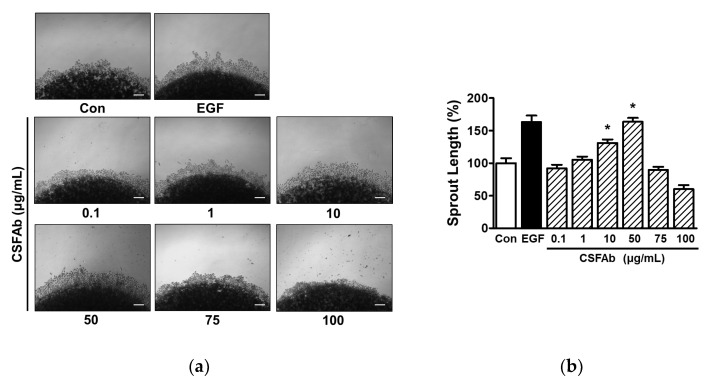
Effect of *Caragana sinica* flower absolute on HaCaT cell sprout formation. (**a**) Representative images of experimental results. HaCaT cells were mixed with collagen, spotted in the wells of a 24-well plate, and treated with or without *Caragana sinica* flower absolute (CSFAb; 0.1–100 µg/mL) for 48 h. Spots and cell sprouts were incubated with Diff-Quick solution and images were taken using a microscope. Recombinant human epidermal growth factor (EGF: 50 µg/mL): positive control. Scale bar = 50 μm. (**b**) Statistical graph of results obtained from panel (**a**). Results are expressed as mean percentages ± SEMs (*n* = 3) of untreated controls (Con). * *p* < 0.05 vs. untreated controls.

It has been reported that collagen sprout outgrowth assays provide a means of assessing the migratory and proliferative activities of keratinocytes [27]. As shown in Figure 2, CSFAb (0.1–100 μg/mL) concentration-dependently increased HaCaT cell sprout outgrowth at 0.1 to 50 μg/mL. Furthermore, this increase in outgrowth was significant at 10 and 50 μg/mL (131.19 ± 5.39% and 163.61 ± 5.92%, respectively, vs. untreated controls) but not at 100 μg/mL (Figure 2). These results demonstrate that CSFAb can induce keratinocyte sprout outgrowth and confirm that CSFAb enhances the migratory and proliferative activities of keratinocytes.

### 2.3. Effects of CS Flower Absolute on Intracellular Signal Proteins in HaCaT Cells

**Figure 3:**
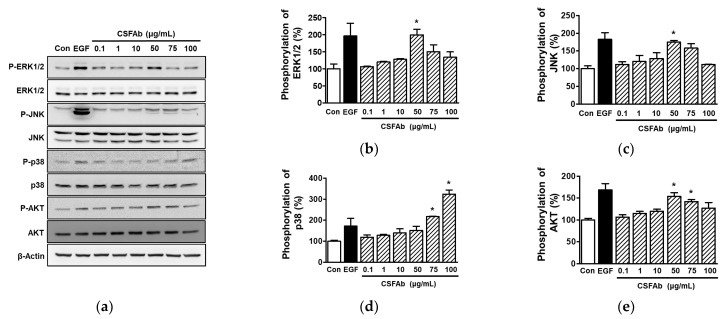
Representative images of immunoblotting results. HaCaT cells were incubated with or without *Caragana sinica* flower absolute (CSFAb; 0.1–100 µg/mL) for 10 min, and cell lysates were immunoblotted with the indicated antibodies. (**b**–**e**) Statistical graphs of phosphorylated ERK1/2 ((**b**) P-ERK 1/2), JNK ((**c**) P-JNK), p38 MAPK ((**d**) P-p38), and AKT expression levels ((**e**) P-AKT) obtained from panel (**a**). Phosphorylation levels are percentages vs. untreated controls (Con). Recombinant human epidermal growth factor (EGF: 5 ng/mL): positive control. Data are expressed as means ± SEMs (*n* = 3/protein). * *p* < 0.05 vs. untreated controls.

To identify the signals that contribute to the migratory and proliferative effects of CSFAb in HaCaT cells, we tested the activation levels in mitogen-activated protein kinases (MAPKs) and serine/threonine-specific protein kinase (AKT), which have been reported to signal migration and proliferation in HaCaT cells [28,29,30]. Immunoblot analysis showed that CSFAb at 50 μg/mL significantly enhanced the phosphorylation of extracellular signal-regulated kinases (ERK1/2) (Figure 3a,b) and c-Jun NH2-terminal kinase (JNK) (Figure 3a,c) (199.96 ± 16.80% and 161.78 ± 16.29%, respectively, vs. untreated controls; Figure 3a–c). In addition, CSFAb at 75 and 100 μg/mL significantly increased p38 MAPK phosphorylation (217.21 ± 2.19% and 324.22 ± 19.54%, respectively, vs. untreated controls; Figure 3a,d) and at 50 and 75 μg/mL increased AKT phosphorylation (151.32 ± 11.80% and 141.93 ± 5.38%, respectively, vs. untreated controls) (Figure 3a,e).

MAPK signaling pathways play crucial roles in controlling diverse cellular events such as differentiation, proliferation, migration, survival, and death [31]. There are three conventional MAPK family members: ERK1/2, JNK, and p38 MAPK [31]. Increased ERK1/2 phosphorylation enhances keratinocyte proliferation and migration [28,32], whereas reduced ERK1/2 phosphorylation has the opposite effect [33,34]. p38 MAPK mediates the proliferative and migratory responses of keratinocytes [29,35]. Furthermore, it has been reported that increased JNK phosphorylation induces migration and proliferation responses in keratinocytes [30] but that JNK does not participate in these processes [35,36]. These reports on JNK imply that it may not be a critical kinase for stimulating keratinocyte proliferation and migration. In the present study, CSFAb increased the activation levels of all three MAPK types in HaCaT cells, suggesting that CSFAb upregulates HaCaT cell migration and proliferation by activating the MAPK signaling pathways. Moreover, AKT signaling can promote cell proliferation, migration, survival, and growth [27,37], and studies have shown that the AKT signaling pathway is implicated in the activation of keratinocyte migration and proliferation [28,32,38]. In this study, CSFAb stimulated AKT activation in HaCaT cells, indicating that CSFAb can induce proliferation and migration via AKT activation. Taken together, it appears that CSFAb promotes the upregulation of keratinocyte proliferation and migration by activating AKT and/or MAPK pathways.

### 2.4. Collagen Synthesis by HaCaT Cells Exposed to CS Flower Absolute

**Figure 4:**
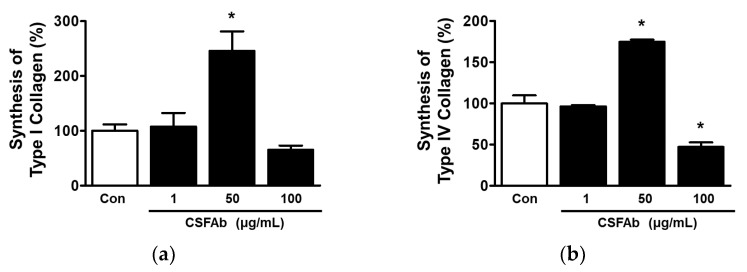
Effects of *Caragana sinica* absolute on type I and type IV collagen syntheses in HaCaT cells. Cells were treated with or without *Caragana sinica* flower absolute (CSFAb; 1–100 µg/mL) for 48 h. Collected conditioned media were treated with anti-type I (*n* = 3; (**a**)) or anti-type IV collagen antibody (*n* = 3; (**b**)) and subjected to sandwich ELISA. Results are expressed as mean percentages ± SEMs of untreated controls (Con). * *p* < 0.05 vs. untreated controls.

To evaluate the effect of CSFAb on collagen synthesis in HaCaT cells, we performed an immunosorbent assay (ELISA) on the conditioned medium (CM) of cells cultured in the presence of CSFAb. Treatment with CSFAb at 50 μg/mL significantly increased the levels of collagens type I and IV in CM of HaCaT cells (245.75 ± 35.83% and 174.75 ± 2.74%, respectively, vs. untreated control CM) (Figure 4a,b).

Skin wrinkles and dryness are typical features of skin aging [2], and thus, substances with moisturizing and anti-wrinkle effects are candidate anti-aging agents. ECM collagens are important for maintaining skin tension, strength, and elasticity [9,10], and reductions in their levels can cause skin wrinkling [39]. In aged skin exposed to UV light, collagen synthesis decreases, and its degradation increases [2,9]. Therefore, regulating collagen content is an effective strategy for reducing wrinkles and preventing wrinkle formation.

Type I collagen is a fundamental structural skin protein and the most abundant collagen. On the other hand, type IV collagen is a major structural basement membrane protein [10]. Keratinocytes in the epidermis produce collagen types I and IV [27,40]. The up-regulation of collagen type I inhibits wrinkle formation in UVB-exposed skin [41]. It has been reported that collagen type IV degradation is increased in UVB-induced wrinkled mouse skin and that the inhibition of collagen type IV degradation reduced wrinkle formation in UVB-irradiated skin [42]. Therefore, it is thought that skin wrinkle formation is linked with low collagen type I and IV levels. In the present study, levels of collagen type I and IV were increased in CM of HaCaT cells treated with CSFAb at 50 μg/mL, implying that CSFAb may increase collagen type I and IV synthesis in keratinocytes. In addition, CSFAb at 50 μg/mL induced an increase in HaCaT cell proliferation. Therefore, increased levels of collagen type I and IV in CM of HaCaT cells may result from an increase in their intracellular biosynthesis and/or an increase in cell number in HaCaT cell response to CSFAb. Taken together, our results indicate that CSFAb might ameliorate skin wrinkles by inducing type I and type IV collagen production by keratinocytes. Thus, CSFAb seems to be a candidate anti-wrinkle treatment.

### 2.5. Effect of CS Flower Absolute on MMP-2 and MMP-9 Activities in HaCaT Cells

**Figure 5:**
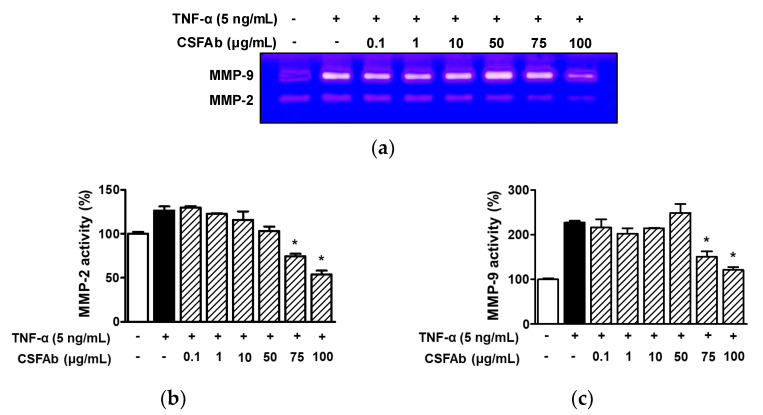
Effect of *Caragana sinica* flower absolute on MMP-9 and MMP-2 activities in HaCaTs exposed to TNF-α. (**a**) Representative image of zymography results. Cells were incubated with or without CSFAb (0.1–100 μg/mL) in the presence or absence of TNF-α (5 ng/mL) for 12 h. Conditioned media were subjected to a gelatin zymography assay to analyze MMP-9 and MMP-2 activities. (**b**,**c**) Statistical graphs of MMP-2 ((**b**) *n* = 2) and MMP-9 ((**c**) *n* = 2) and levels are shown in panel (**a**). Data are expressed as percentages of cells in the quiescent state. * *p* < 0.05 vs. the quiescent state.

Gelatin zymography was performed on the CM of HaCaT cells treated with CSFAb (0.1–100 μg/mL) in the presence of TNF-α (5 ng/mL). TNF-α at 5 ng/mL enhanced the activity of MMP-2 in HaCaT cells (126.47 ± 4.56% vs. untreated controls), and this response was attenuated by CSFAb at 75 and 100 μg/mL (74.50 ± 2.73% and 53.87 ± 4.23%, respectively, of the untreated control level) (Figure 5a,b). TNF-α (5 ng/mL) increased the activity of MMP-9 (227.13 ± 4.12% of untreated controls) and was significantly decreased by treatment with CSFAb at 75 and 100 μg/mL (150.94 ± 11.88% and 121.10 ± 6.21%, respectively, of untreated controls) (Figure 5a,b). However, CSFAb did not significantly change the activity of MMP-2 or -9 in HaCaT cells at concentrations in the range of 0.1 to 50 μg/mL (Figure 5).

Low ECM collagen levels are closely associated with wrinkle formation in aged skin [39], and ECM collagens are degraded by MMPs. MMP-2 and MMP-9 can impair basement membrane and epidermis characteristics by degrading type I and II collagen [14]. Furthermore, kinetic studies have shown that MMP-2 and -9 degrade collagen more effectively than other MMPs [43]. MMP-2 can degrade collagen type I and IV, whereas MMP-9 digests collagen IV more effectively than collagen type I [14]. MMPs can be activated by growth factors, cytokines, UV light, and aging and are produced and secreted by keratinocytes and fibroblasts [14]. Furthermore, the proinflammatory cytokine TNF-α can induce MMP-2 and -9 in keratinocytes [44]. We found that TNF-α upregulated the activities of both in HaCaT cells and that these upregulations were attenuated by CSFAb. Furthermore, levels of activated MMP-2 and MMP-9 were elevated in the extracts of mouse skins wrinkled by UVB, and these increases were suppressed by inhibiting the activities of MMP-2 and -9 [42]. In another study, MMP-9 inhibition reduced wrinkle formation in UVB-exposed skin [45]. These reports indicate that reducing the activities of MMP-2 and MMP-9 has negative effects on wrinkle formation. Therefore, our results suggest that CSFAb may have inhibitory effects on MMP2 and/or MMP-9 and suppress skin wrinkle formation.

### 2.6. Effect of CS Flower Absolute on Hyaluronic Acid in HaCaT Cells

**Figure 6:**
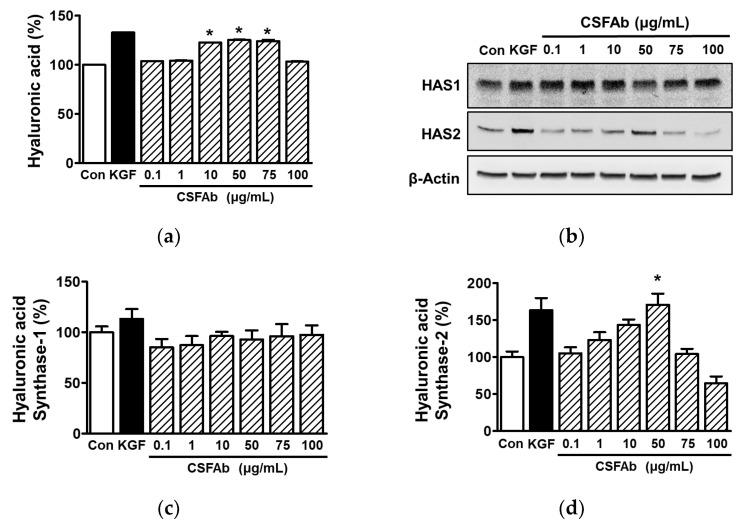
Effects of *Caragana sinica* flower absolute on hyaluronic acid synthesis in HaCaT cells. (**a**) Hyaluronic acid levels. HaCaT cells were incubated with or without *Caragana sinica* flower absolute (CSFAb; 0.1–100 µg/mL) for 12 h, and conditioned media were analyzed using an ELISA kit (*n* = 3), as described in Section 3. (**b**–**d**) Expression levels of hyaluronic acid synthases (HASs). HaCaT cells were incubated with or without *Caragana sinica flower* absolute (CSFAb; 0.1–100 µg/mL) for 12 h, and lysates were immunoblotted with HAS-1, HAS-2, HAS-3, or β-actin antibodies (*n* = 3/protein). Recombinant keratinocyte growth factor (KGF: 20 ng/mL): positive control. (**b**) Representative images of results. (**c**,**d**) Statistical graphs of HAS-1 (**c**) and HAS-2 (**d**) levels in panel (**a**). Results are expressed as mean percentages of untreated controls (Con). * *p* < 0.05 vs. untreated controls.

HA is a key contributor to skin moisture retention and thus reduces skin wrinkling [20]. Keratinocytes were used to analyze HA synthesis related to skin moisturizing ability [46]. To determine if CSFAb affects HA levels, we examined the effect of CSFAb on HA synthesis in HaCaT cells. Treatment of HaCaT cells with CSFAb (0.1–100 μg/mL) significantly increased HA levels in CM at concentrations of 10 to 75 μg/mL (Figure 6a). In addition, treatment with CSFAb at concentrations of 0.1 to 100 μg/mL did not affect HAS-1 expression in HaCaT cells (Figure 6b,c). However, CSFAb (0.1 to 50 μg/mL) concentration-dependently upregulated HAS-2 expression in HaCaT cells, and this peaked at 50 μg/mL (159.30 ± 14.08% of the untreated control level) (Figure 6b,d). On the other hand, our result showed that HAS-3 expression was not detected in HaCaT cells regardless of CSFAb treatment.

HA can bind water molecules, which is important for retaining moisture and maintaining skin elasticity [20]. It has been reported that low HA levels in skin are associated with water loss and wrinkle formation [46], and that topical application of HA reduced the wrinkling of aged skin [47]. HAS synthesizes HA and has three isoforms, viz., HAS-1, -2, and -3, in mammals [20]. These reports indicate that increased levels of HA and HAS can reduce wrinkling by increasing water levels in skin. We found that CSFAb increased HA contents and HAS-2 expression in HaCaT cells, which indicates that CSFAb upregulates HA production by increasing HAS-2 expression in keratinocytes and suggests that CSFAb may have the ability to promote moisture levels in the epidermal layer.

### 2.7. Chemical Composition of ITMFAb

**Figure 7:**
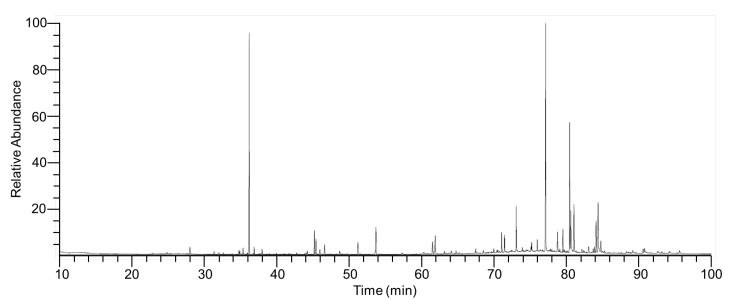
GC/MS total ion chromatogram of *Caragana sinica* flower absolute showing the peaks of the 46 identified compounds listed in Table 1.

The composition of CSFAb was analyzed by GC/MS, which revealed the presence of 46 compounds (Table 1, Figure 7). The four most abundant compounds were hexatriacontane (18.73%), methyl undecanoate (12.88%), 15-nonacosanol (11.77%), and nonacosane (19.12%). The peak area percentages of the next eight most abundant components were as follows: palmitic acid TMS derivative (3.63%), octacosane (3.48%), neophytadiene (3.33%), ethyl linolenate (2.64%), clionasterol (1.71%), icosanal (1.65%), 2,2′-methylenebis(4-methyl-6-tert-butylphenol) (1.56%), and ethyl linoleate (1.53%) (Table 1).

Among the identified compounds, nonanal has been reported to promote the proliferation and migration of human hair follicle dermal papilla cells [48] and lupeol was reported to increase keratinocyte migration [49]. Thus, it may be that these two compounds contributed to the CSFAb-induced migration and proliferation of keratinocytes observed in the present study. In addition, CSFAb was also found to contain several compounds that can promote skin wound healing responses. For example, lupeol has been reported to have anti-inflammatory effects and to promote angiogenesis and growth factor production [50]. Phytol, linolenic acid, and ethyl linoleate are the main components of extracts from many plants and have anti-inflammatory, hair and pigment recovery, angiogenic, and/or wound healing effects [51,52,53,54]. Furthermore, linolenic acid and phytol have been previously reported to have inhibitory effects on skin wrinkling-related responses, such as collagen synthesis and water loss [55,56,57,58]. To summarize, several components of CSFAb have been suggested to have the ability to heal skin wounds or reduce skin wrinkling. We recommend that further studies be undertaken to explore the effects of the identified CSFAb compounds on wound healing-associated responses, MMP activity, and collagen and HA synthesis in keratinocytes.

## 3. Materials and Methods

### 3.1. Materials

Phosphate-buffered saline (PBS) and Dulbecco’s modified eagle medium (DMEM) were purchased from Welgene (Daegu, Republic of Korea) and the EZ-CyTox kit was from DAEIL LAB Service (Seoul, Republic of Korea). FBS, penicillin/streptomycin (P/S), and trypsin-ethylenediamine tetra-acetic acid (EDTA) were purchased from Gibco BRL (Gaithersburg, MD, USA). Dimethyl sulfoxide (DMSO) was purchased from MilliporeSigma (St. Louis, MO, USA), and recombinant keratinocyte growth factor (KGF), recombinant human EGF (purity > 97%), and tumor necrosis factor-α (TNF-α) were from R&D Systems (Minneapolis, MN, USA). Bovine serum albumin (BSA) and type I collagen were obtained from BD Bioscience (Franklin Lakes, NJ, USA). The antibodies used were anti-rabbit IgG, anti-mouse IgG, anti-AKT, anti-phospho AKT, anti-JNK, anti-phospho JNK, anti-p38 MAPK, anti-phospho p38 MAPK, anti-ERK1/2, anti-phospho ERK1/2 (Cell Signaling, Beverly, MA, USA), anti-HAS-1, anti-HAS-2, anti-HAS-3 (Novus Biologicals, Littleton, CO, USA), polyclonal anti-type I and IV collagens, monoclonal anti-type I and IV collagens (Abcam, Cambridge, UK), and β-actin (MilliporeSigma, Burlington, MA, USA).

### 3.2. Preparation of Caragana Sinica Flower Absolute

CS samples were obtained from the field near Hanaro Farm (Haenam County, Jeollanam-do, Republic of Korea) (34°23′00.4″ N 126°33′59.0″ E; 25 April 2019). CS identity was confirmed by Jong-Cheol Yang from the Division of Forest Biodiversity and Herbarium, Korea National Arboretum (Republic of Korea). A voucher specimen (no. CS-0001) was kept at the Herbarium of the College of Life and Health Science, Hoseo University (Republic of Korea). Absolute was extracted from CS flowers by solvent extraction as previously described [27]. In brief, flowers (5.27 kg) were completely immersed in hexane (Samchun, Pyeongtaek, Republic of Korea) at room temperature (RT) for 1 h. Solvent was removed from extracts using a rotary evaporator (EYELA, Tokyo, Japan) at 25 °C under vacuum to yield a dark yellow waxy residue (concrete), which was mixed with ethanol and left at −20 °C overnight. The mixture was then filtered through a sintered glass funnel, and the ethanol was evaporated at 35 °C. The light yellow wax obtained (absolute) (CSFAb; 1.50 g, yield 0.028%, *w*/*w*) was stored at −80 °C until required.

### 3.3. Analysis and Identification of Compounds from Caragana sinica Flower Absolute

Compounds in CSFAb were analyzed and identified by GC/MS at the National Instrumentation Center for Environmental Management (NICEM, Seoul National University, Republic of Korea). GC/MS data were acquired using a TRACE 1310 GC unit attached to an ISQ LT single quadrupole mass spectrometer (Thermo Scientific, Waltham, MA, USA) as previously described [59]. Briefly, derivatized CSFAb was separated on a DB-5MS column (60 m × 0.25 mm, 0.25 μm; Agilent Technologies, Santa Clara, CA, USA) at a flow rate of 1 mL/min. The GC oven temperature was programmed as follows; 50 °C for 5 min, 50 to 65 °C at 10 °C/min, 65 to 210 °C at 5 °C/min, 210 to 310 °C at 20 °C/min, and held at 310 °C for 10 min. The scanned mass range was 35–550 m/z, and the data acquisition rate was 0.2 scans/s in the MS setting. The transfer line and ion source temperatures were 300 °C and 270 °C, respectively. Metabolites were identified by comparing their MS spectra and retention indices (RI) with reference standards obtained from the NIST/NIH/EPA mass spectral library (NIST 11, version 2.0 g). A standard mixture of n-alkanes (C_7_–C_30_) was used to determine compound RIs. Final identification was performed by comparing retention times and spectra with those of commercially available standards.

### 3.4. Cell Culture

The human keratinocyte cell line HaCaT (human, adult, low-calcium, high-temperature keratinocytes) cells were provided by the Daegu Gyeongbuk Institute for Oriental Medicine Industry (Daegu, Gyeongsan City, Republic of Korea). Cells were maintained in DMEM containing FBS (10%) and 1% P/S (1%) and incubated in a humidified 95% air/5% CO_2_ atmosphere at 37 °C. Cells were cultivated to 70–80% confluence for each experiment.

### 3.5. Cell Viability Assays

HaCaT cell viability was measured using a WST assay (the EZ-CyTox kit) as previously described [59]. HaCaT cells were seeded into 96-well cell culture plates with 5 × 10^3^ cells per well, treated with different concentrations of CSFAb for 48 h in a humidified 95% air/5% CO_2_ atmosphere at 37 °C, and then with EZ-CyTox reagent (10 μL/well) for 30 min under the same conditions. Cell viability levels were determined by measuring absorbance at 450 nm using a multi-well plate reader (Synergy 2, Bio-Tek Instruments, Winooski, VT, USA) and expressed as percentages of untreated controls. The concentration range used for subsequent experiments was determined using the results obtained.

### 3.6. Proliferation Assays

Cell proliferation was evaluated using a BrdU incorporation assay kit (Roche, Indianapolis, IN, USA) as previously described [59]. Briefly, HaCaT cells were plated in a 96-well plate with 2 × 10^3^ cells per well and were treated with various concentrations of CSFAb dissolved in DMEM containing 0.5% DMSO or a positive control EGF (50 ng/mL) for 36 h. BrdU labeling solution (10 μM) was then added and incubated at 37 °C for 12 h. The cells were then fixed, to denature DNA, with FixDenat solution from the BrdU kit for 30 min at RT, followed by incubation with peroxidase-labeled anti-BrdU monoclonal antibody at RT for 90 min. BrdU antibody complexes were detected using a luminometer (Synergy 2; Bio-Tek Instruments). Cell proliferation levels were expressed as percentages of those of untreated controls.

### 3.7. Migration Assay

Cell migration was assessed using a 48-well Boyden microchemotaxis chamber (Neuro Probe Inc., Gaithersburg, MD, USA) as in a previous report [59]. Lower chambers were loaded with different concentrations of CSFAb or the positive control EGF (1 ng/mL) in DMEM containing 0.1% BSA. A membrane coated with type Ι collagen was laid over media in lower chambers. Upper chambers were loaded with HaCaT cells at a density of 5 × 10^4^ cells per well in DMEM supplemented with 0.1% BSA. Lower and upper chambers were then assembled and incubated at 37 °C for 4 h when the membrane was fixed and stained using Diff-Quick solution (Baxter Healthcare, Miami, FL, USA). Cells that migrated to the lower surface of the membrane were counted using an optical microscope at ×200. Levels of cell migration were presented as percentages of those of untreated controls.

### 3.8. Collagen Sprout Outgrowth Assay

A collagen sprout outgrowth assay was performed to simultaneously assess the migration and proliferation of HaCaT cells [27]. In brief, HaCaT cells (2.5 × 10^7^ cells/mL) were added to a solution of type I collagen, 10× DMEM, and 1N NaOH (pH 7.2). This mixture was spotted (2.5 × 10^5^ cells/10 μL) into the wells of a 24-well culture plate and dried. Spots were then incubated with various concentrations of CSFAb or the positive control (EGF, 50 ng/mL) at 37 °C for 48 h, and spots and sprouts were fixed and stained using Diff-Quick solution (Baxter Healthcare). Images of spots and sprouts were obtained using an optical microscope at ×100, and sprout lengths were analyzed using Scion Image software (Frederick, MD, USA). Sprout outgrowth levels are expressed as percentages of the sprout outgrowths of untreated controls.

### 3.9. Collagen Synthesis Assay

Levels of collagen synthesis by keratinocytes were confirmed using an ELISA as in previous report [27]. In brief, HaCaT cells were seeded in 100 mm cell culture dishes with 5 × 10^5^ cells per well and treated with various concentrations of CSFAb for 48 h at 37 °C. Collected media were then centrifuged sequentially at 500× *g*, 800× *g*, or 1000× *g* for 10 min, and 100 μL of supernatants (conditioned media) were added to each well of a 96-well microtiter plates coated with type I or IV collagen monoclonal antibodies used as capture antibodies, and treated with biotin-conjugated collagen type I or IV polyclonal antibody (diluted 1:2000 in 1% BSA/PBS) for 90 min at RT. The wells were then washed with PBS and treated with streptavidin–horseradish peroxidase conjugate (Roche, Indianapolis, IN, USA) (diluted 1:5000 in 1% BSA/PBS) for 1 h at RT. After washing wells with PBS, they were treated with ECL (enhanced chemiluminescence) solution (Thermo Fisher Scientific, Waltham, MA, USA). Luminescence was detected using a luminometer (Synergy 2, Bio-Tek Instruments), and the results are presented as percentages of untreated controls.

### 3.10. Immunoblotting

Lysates of cells lysed in radioimmunoprecipitation buffer (Cell Signaling) were centrifuged (17,000× *g*, 15 min, 4 °C) and supernatants were collected. Protein concentrations in supernatants were estimated using DC protein assay reagents (Bio-Rad Laboratories, Hercules, CA, USA). Proteins (50–130 μg/lane for kinases or 60–100 μg/lane for hyaluronic acid synthase) were separated by 10% or 12% SDS-PAGE and transferred at 4 °C to polyvinylidene fluoride membranes. Membranes were blocked with 3% non-fat dry milk blocking solution at RT for 2 h, washed with PBS supplemented with 0.05% Tween-20, incubated with target primary antibodies (diluted 1: 1000–10,000), and then with horseradish peroxidase-conjugated secondary antibody at RT for 1 h. Immunoreactive bands were developed using a chemiluminescence substrate and the images were captured using a chemiluminescence imaging system (LuminoGraph, ATTO, Tokyo, Japan). Band densities were determined using Quantity One software (Bio-Rad, Hercules, CA, USA). The expression levels of phosphorylated kinases and HAS are presented as percentages of total protein in non-treated controls. β-actin was used as the internal control

### 3.11. Hyaluronic Acid Synthesis Assay

HaCaT cells (3 × 10^5^ cells/well) were incubated in a 6-well plate for 12 h and then starved for 6 h in serum-free DMEM to eliminate any effect of FBS. Cells were then incubated with different concentrations of CSFAb in 2 mL of serum-free DMEM for 12 h, and media were then collected and centrifuged at 500× *g*, 800× *g*, or 1000× *g* for 10 min. HA levels in supernatants (conditioned media; 50 μL/well) were determined using an ELISA kit (R&D Systems). HA levels were measured using a multi-well plate reader (Synergy 2) at 450 nm.

### 3.12. Gelatin Zymography Assay

MMP-2 and MMP-9 activities in HaCaT cells were determined using a gelatin zymography assay [60,61]. Cells (1 × 10^6^ cells/mL) were seeded in a 100 mm cell culture dish and then were treated with or without TNF-α (5 ng/mL) in the presence or absence of different concentrations of CSFAb for 24 h at 37 °C. Collected cell media were centrifuged sequentially at 500× *g*, 800× *g*, and 1000× *g* for 10 min. Supernatants (conditioned media) were mixed with 5× sample buffer solution, incubated for 1 h at 37 °C, and loaded into 10% SDS polyacrylamide gels containing 0.5% gelatin (MilliporeSigma) and electrophoresed. Gels were washed in 2.5% Triton X-100 for 1 h, incubated with reaction buffer solution for 20 h at 37 °C, stained with 0.05% Coomassie Brilliant Blue R-250 (Bio-Rad), and bleached. Relative band densities were determined using an imaging scanner (SL-M4080FX, Samsung, Suwon, Republic of Korea). Gelatinolytic activities were normalized vs. total protein levels.

### 3.13. Statistical Analysis

The analysis was performed using Student’s *t*-test for comparisons between pairs of groups and by one-way analysis of variance (ANOVA) followed by Tukey’s post hoc test for multiple comparisons using GraphPad Prism (version 5.0; Graphpad Software, Inc., LaJolla, CA, USA). Results are presented as means ± standard errors, and *p* values < 0.05 were considered significant.

## 4. Conclusions

CSFAb contained forty-six components and promoted the proliferation, migration, and sprout outgrowth of HaCaT cells. In addition, CSFAb increased the activation of MAPKs (ERK1/2, JNK, p38 MAPK) and AKT and the levels of HA, HAS-2, and collagens type I and IV, but decreased the TNF-α-induced activities of MMP-2 and MMP-9. These findings indicate that CSFAb may have positive effects on wound healing by increasing keratinocyte migration and proliferation and on skin wrinkle improvement by increasing collagen synthesis, decreasing collagen degradation, and promoting hydration in keratinocytes. Thus, our findings suggest that CSFAb offers a potential developmental starting point for pharmaceuticals or cosmetics that promote wound healing and have beneficial effects on skin anti-wrinkling. However, it may be necessary in future studies to further identify the key bioactive components of CSFAb which promote skin wound healing and anti-wrinkle-associated activities, and to investigate its activities in in vivo or other skin cells.

## Figures and Tables

**Table 1:** Components of the absolute of *Caragana sinica* flowers.

No	Component Name	RT ^1^	RI ^2^	Area (%)	CAS No.
1	Cyclopentanone, 2-sec-butyl-	24.8	1237	0.18	6376-92-7
2	Nonanal	27.96	1257	0.63	124-19-6
3	Trimethylsilyl nonanoate	34.73	1300	0.21	82326-11-2
4	2,2,4-Trimethyl-1,3-pentanediol diisobutyrate	34.86	1301	0.24	6846-50-0
5	Propanoic acid, 2-methyl-, 3-hydroxy-2,2,4-trimethylpentyl ester	35.3	1305	0.36	77-68-9
6	Methyl undecanoate	36.16	1312	12.88	1731-86-8
7	Farnesane	36.84	1318	0.48	3891-98-3
8	2-Allyl-5-t-butylhydroquinone	37.94	1327	0.42	73685-60-6
9	2-Nonadecanone	44.18	1380	0.45	629-66-3
10	Neophytadiene	45.16	1388	3.33	504-96-1
11	3,7,11,15-Tetramethyl-2-hexadecen-1-ol	45.92	1395	0.51	102608-53-7
12	Phytol	46.58	1401	0.75	150-86-7
13	Ethyl palmitate	51.18	1469	1.38	628-97-7
14	Palmitic Acid, TMS derivative	53.67	1506	3.63	55520-89-3
15	Heneicosane	57.28	1551	0.30	629-94-7
16	Linolenic acid	60.29	1589	0.45	463-40-1
17	Ethyl linoleate	61.48	1607	1.53	544-35-4
18	Ethyl linolenate	61.87	1614	2.64	1191-41-9
19	Ethyl stearate	63.14	1639	0.30	111-61-5
20	α-Linolenic acid, TMS derivative	64.1	1658	0.69	97844-13-8
21	1,2-Epoxyhexadecane	64.75	1671	0.36	7320-37-8
22	Pentatriacontane	67.47	1732	0.48	630-07-9
23	2,2-Dideutero octadecanal	68.51	1760	0.24	56555-07-8
24	1-Heptatriacontanol	69.96	1799	0.36	105794-58-9
25	Ethyl arachidate	70.4	1813	0.39	18281-05-5
26	2,2′-Methylenebis(4-methyl-6-tert-butylphenol)	71.05	1835	1.56	119-47-1
27	Octadecanal	71.44	1848	1.44	638-66-4
28	Octacosane	73.06	1902	3.48	630-02-4
29	Bis(2-ethylhexyl) phthalate	73.9	1934	0.45	117-81-7
30	Heptacosane	75.19	1984	1.11	593-49-7
31	Tetracosanal	75.96	2013	0.84	57866-08-7
32	Hexatriacontane	77.13	2058	18.73	630-06-8
33	1,3-bis[(4′-Oct-7′-en-1′-yl)phenyl]-prop-2-yn-1-ol	77.78	2082	0.21	None
34	Icosanal	79.51	2140	1.65	2400-66-0
35	Nonacosane	80.46	2169	19.12	630-03-5
36	Dotriacontane	82.14	2217	0.39	544-85-4
37	4-[4′-Acetylphenyl]-2-methyl-6-(1′-hydroxy-1′-methylpropyl)pyrido[3,4-c]thiazole	83.05	2240	0.81	None
38	17-Pentatriacontene	83.69	2256	0.33	6971-40-0
39	15-Nonacosanol	84.37	2272	11.77	2764-81-0
40	Ethyl iso-allocholate	88.31	2413	0.63	None
41	HAHNFETT	89.17	2470	0.90	None
42	Clionasterol	90.8	2606	1.71	83-47-6
43	beta-Amyrin	92.64	2918	0.39	559-70-6
44	Lupenone	93.18	3014	0.18	1617-70-5
45	Lupeol	94.22	3191	0.42	545-47-1
46	24-Methylenecycloartanol	95.61	3641	0.66	1449-09-8
Total Identified (%)				100.00	

^1^ RT: retention time, ^2^ RI: retention indices. RTs and RIs were determined using a DB-5MS capillary column.

## Data Availability

Not applicable.
